# Estimating International Migration Flows for Pacific Island Countries: A Research Brief

**DOI:** 10.1007/s11113-022-09737-z

**Published:** 2022-08-14

**Authors:** Qing Guan, James Raymer, Juliet Pietsch

**Affiliations:** 1 School of Demography, Australian National University, Canberra, Australia; 2 School of Government and International Relations, Griffith University, Brisbane, Australia

**Keywords:** International migration, Pacific Island countries, Generation-distribution model, Migration flow, Migration data

## Abstract

International migration is an important source of population change and economic development for Pacific Island countries. Migration from the Pacific Island region contributes to labour recruitment in countries like Australia, New Zealand and the United States. However, there are substantial gaps in the understanding of overall migration patterns in this region, impeding the development of relevant policies. In the absence of good migration statistics, we propose and present an alternative approach to examining the levels of migration in the Pacific Island region using model-based estimates. Three sets of recently produced migration flow estimates are consulted to explore the immigration and emigration levels and key migration corridors in the Pacific Island region between 2000 and 2019. Where reported migration statistics are available, we evaluate the performance of model-based estimates and highlight if there are problems with the reported data. This research brief demonstrates the value of model-based estimates to inform migration in the Pacific Island region.

## Introduction and Background

The Pacific Island region has a population of 12.6 million, comprising of 11.3 million residents in Melanesia, 677 thousand in Polynesia, and 546 thousand in Micronesia (Pacific Data Hub, 2021).﻿^1^ Migration across the islands has had a long history going back centuries. More recently, colonial legacies and family and social ties have established pathways of migration between the Pacific Islands and New Zealand, the United States and Australia for temporary and permanent settlement (McKenzie, 2007, Table 6.1; Ware, 2005; Lee, 2009). Many Pacific Island countries have large proportions of their citizens living abroad (McKenzie, 2007). Before the COVID-19 pandemic, United Nations (2019) estimated that there were 751 thousand Pacific Islanders living outside their country of birth with 310 thousand living in the Pacific Island region.

Countries that receive large numbers of Pacific Islanders are facing population ageing and shortages of skilled and unskilled workers (Chand et al., 2021). As such, numerous schemes have encouraged migration from Pacific Island countries to move to New Zealand, the United States and Australia. Due to historical colonial ties, Pacific Islanders from the Federated States of Micronesia, Palau and the Republic of the Marshall Islands have visa-free access to the United States through the Compact of Free Association. Pacific Islanders from the Cook Islands, Niue and Tokelau have visa-free access to New Zealand. In New Zealand, the Pacific Access Category scheme grants permanent residency each year to migrants from Fiji, Kiribati, Tonga and Tuvalu (Ash & Campbell, 2016, p. 54). Once permanent residence or citizenship is established in New Zealand, migrants can enter Australia through the Trans-Tasman Travel Arrangement (Vasta, 2004).

Since 2009, New Zealand and Australia have increased their seasonal worker programmes to fulfil labour market shortages. In 2007, New Zealand introduced a Recognized Seasonal Employer scheme to allow employers from the horticulture and viticulture industries to recruit seasonal workers from Pacific Island countries. The cap for the programme increased from 5000 in 2007 to 16,000 in 2021–2022 (New Zealand Immigration, 2022). In 2018, Australia introduced the Pacific Labour Scheme which recruits workers from up to 10 Pacific Island countries for up to 3 years. During the COVID-19 pandemic in 2021, Australia accepted 12 thousand workers from the Pacific Islands under the Seasonal Worker Programme and Pacific Labour Scheme (Courtney, 2021). The seasonal worker migration programmes have raised several concerns, including the higher costs associated with recruiting migrants (Bedford et al., 2017), limited options for permanent residency (Howe et al., 2019, p.220), and reports of exploitation, intimidation and wage theft (Fair Work Ombudsman, 2021).

In addition to labour recruitment, there has been a growing international awareness of the need to incorporate Pacific Islands migration into the development and aid programmes of major destination countries. Voluntary migration is viewed as a potential mitigation strategy to reduce population pressure (including high youth unemployment rates, see International Labour Organization (ILO), 2018, 2021) and increase environmental sustainability. It may also be used to increase income diversification through migrant remittances, as many in the Pacific Islands live on subsistence agriculture and fishing. Finally, migration can operate to enhance the education and technology skills in the Pacific Islands through skills transfers (Ash & Campbell, 2016, p. 58).

In order to assess the effects of migration on development and to devise relevant policies, information on the migration patterns and trends are needed. However, migration flow statistics and migrant population data^2^ are poorly documented and reported for Pacific Island countries (Campbell & Warrick, 2014; Raymer et al., 2022). Many Pacific Island countries lack the capacity to collect and/or analyse migration data from departure and arrival cards or national censuses (Campbell & Warrick, 2014). Some major receiving countries, including Australia and New Zealand, have information on the migration flows but there are substantial gaps in our understanding of overall migration patterns, including migration amongst Pacific Island countries (Curtain & Howes, 2020; United Nations, 2015b).

In this research brief, we explore international migration in the Pacific Island region over time using recently produced international migration estimates covering flows from, to and between 53 Asia and Pacific Island populations (Raymer et al., 2022). In these estimates, migration flows include all persons who move across national borders for the purpose of changing their country of usual residence, in line with United Nations Recommendations (1998, 2020). These estimates are compared with two other model-based estimates (Abel, 2018; Azose & Raftery, 2019). The analyses presented in this research brief focus on the migration patterns estimated for the Pacific Island region, where hardly any reliable official statistics are available.

## Data Situation and Models

### Data Availability

Migration flow statistics for Pacific Island countries are practically non-existent, except for statistics reported by major destination countries and incomplete emigration numbers reported by some Pacific Island countries. Migration between Pacific Island countries and Australia, New Zealand and the United States is available from the United Nations (2015a) database on *International Migration Flows to and from Selected Countries* (IMFSCD). In IMFSCD, definitions of migrants differ by reporting country. In the United States, immigrants are foreigners granted permanent residence status in a particular fiscal year. In Australia, immigrants (emigrants) are measured as persons entering (departing) the country and remaining for 12 months out of 16 months. In New Zealand, immigrants are those who remain for 12 months or longer. Australia and New Zealand also publish visa category statistics, which include seasonal workers for citizens of several Pacific Island countries. However, details on their duration of stay and previous or next place of usual residence are not available.

Data reported by Pacific Island countries can be retrieved from the International Labour Migration Statistics (ILMS) database and the Pacific Data Hub (2022). ILMS data can be accessed from ILOSTAT (ILO, 2022) and ILMS Database in ASEAN (ILO, 2015). ILMS focuses on international migrant workers and has data reported by two Pacific Island countries: Samoa and Fiji (for selected years between 2010 and 2020). Pacific Data Hub has annual international arrivals and departures statistics from Tonga (for 2005–2017). However, users are not able to distinguish long-term migrants from tourists in the Tongan publications.

### Modelling International Migration Flows

In the scarcity of reported international migration flows, efforts have been made to estimate migration flows based on United Nations’ migrant stock data. An advancement in this direction is the flow-from-stock method used to obtain 5-year migration flow estimates (Abel, 2013). In this research brief, we examine two recent sets of international migration flow estimates using this method: Abel (2018) and Azose and Raftery (2019) for the 5-year periods 2000–2005, 2005–2010 and 2010–2015, available from the publications’ online data repository. We also include a third set of migration flow estimates representing annual flows from 2000 to 2019 produced by Raymer et al. (2022). This approach uses a range of covariate information to first generate emigration flows, which are then distributed across possible destination﻿s, i.e. a generation-distribution model framework (Willekens, 1994; Willekens & Baydar, 1986). The estimates are available from the publication’s online data repository.

## Estimated International Migration Patterns

### Total Migration Levels

**Fig. 1:**
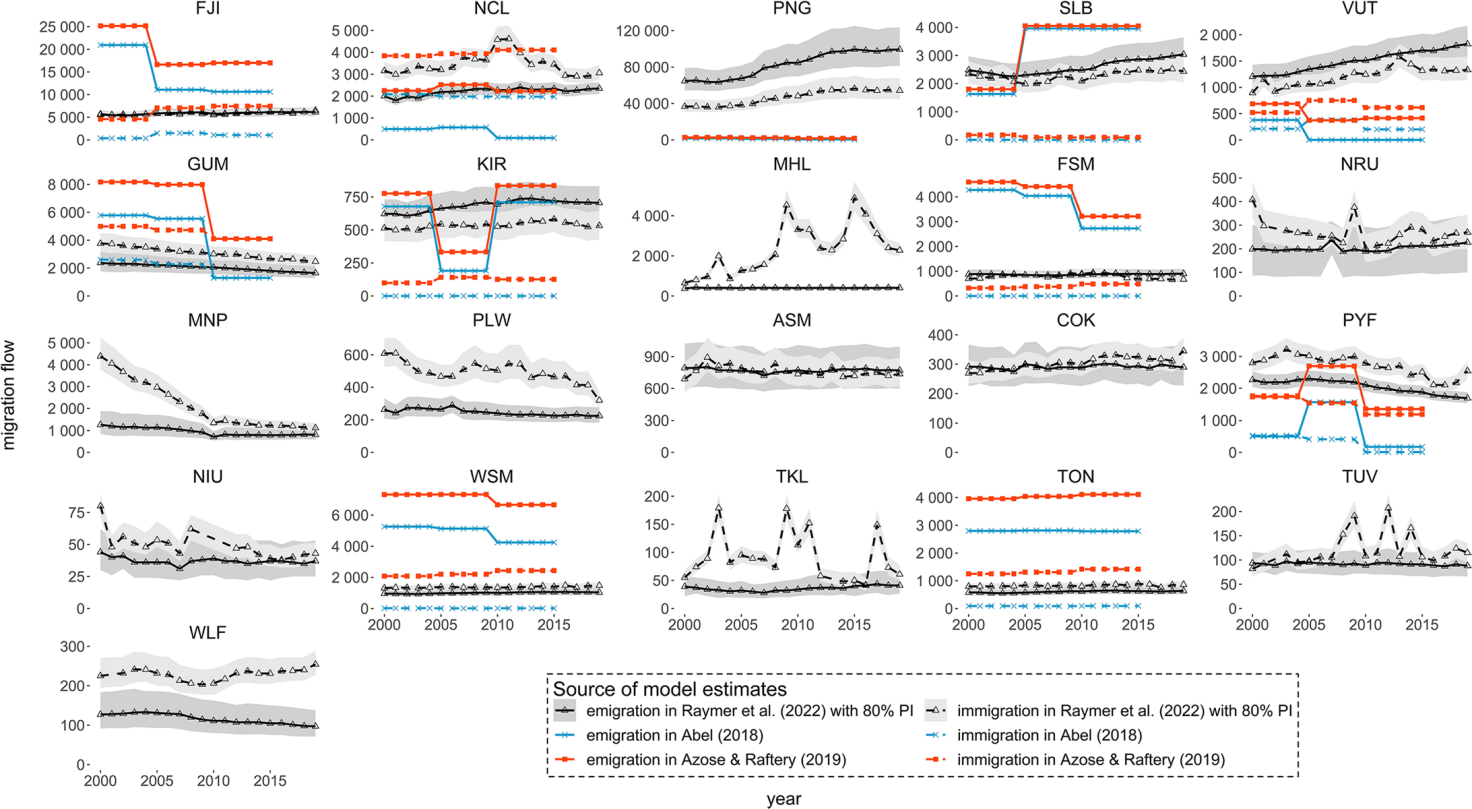
Estimated total immigration and emigration to and from Pacific Island countries: 2000–2019. Note: Immigration to TUV in 2001–2002, WLF in 2001, NRU in 2002, NIU in 2009–2011 and TKL in 2013 from Raymer et al. (2022) are excluded for they include extreme values. Country code-names correspondence is in the Appendix

Three model-based estimates of immigration and emigration for Pacific Island countries are presented in Fig. 1. ISO3 codes are used to represent countries. Note, to approximate annual flows, the 5-year flow estimates from Abel (2018) and Azose and Raftery (2019) have been divided by three. This assumption comes from Rogers et al. (2003), who compared 5-year and 1-year internal migration proportions in the United States.

Immigration levels in Pacific Island countries range between 0 immigrants (Abel’s model: the Federated States of Micronesia and Kiribati 2000–2015, Solomon Islands 2005–2015) and 56,332 immigrants (Raymer et al.’s model: Papua New Guinea 2015). Emigration levels from Pacific Island countries range between 0 emigrants (Abel’s model: Vanuatu 2005–2015) and 99,416 emigrants (Raymer et al.’s model: Papua New Guinea 2015). Estimates from Abel (2018) and Azose and Raftery (2019) exhibit similar over-time trends between 2000 and 2015 because they used the same model inputs from the United Nations (2015a, 2015b) birthplace-specific migrant stock data.^3^ The Raymer et al. (2022) estimates, on the other hand, are based on modelled propensities to emigrate and have had correlation structures included to ensure return migration.

There are two main advantages of using model-based estimates to inform levels of international migration for Pacific Island countries. First, model-based estimates overcome the absence of reported international migration flow statistics in the Pacific region. With the three sets of model-based estimates, immigration and emigration levels are available for 21 Pacific Island countries, as shown in Fig. 1. In comparison, only three countries have reported migration flows statistics: Fiji, Samoa and Tonga.

The second advantage of model-based estimates is the ability to compare patterns across countries. Missing and inconsistently defined data have been a long-standing challenge in cross-country comparisons of international migration (Abel, 2018, p. 842). In the reported Pacific Island statistics, the definitions of international migrants vary significantly. Definitions reported by Fiji, Samoa and Tonga include nationals employed abroad, emigration of nationals and passengers arrivals and departures (including tourists). In comparison, the three sets of model-based estimates each adopt a consistent definition of international migrants. It enables users to make sense of comparing relative levels of migration flows across countries.

### Key Migration Corridors

**Fig. 2:**
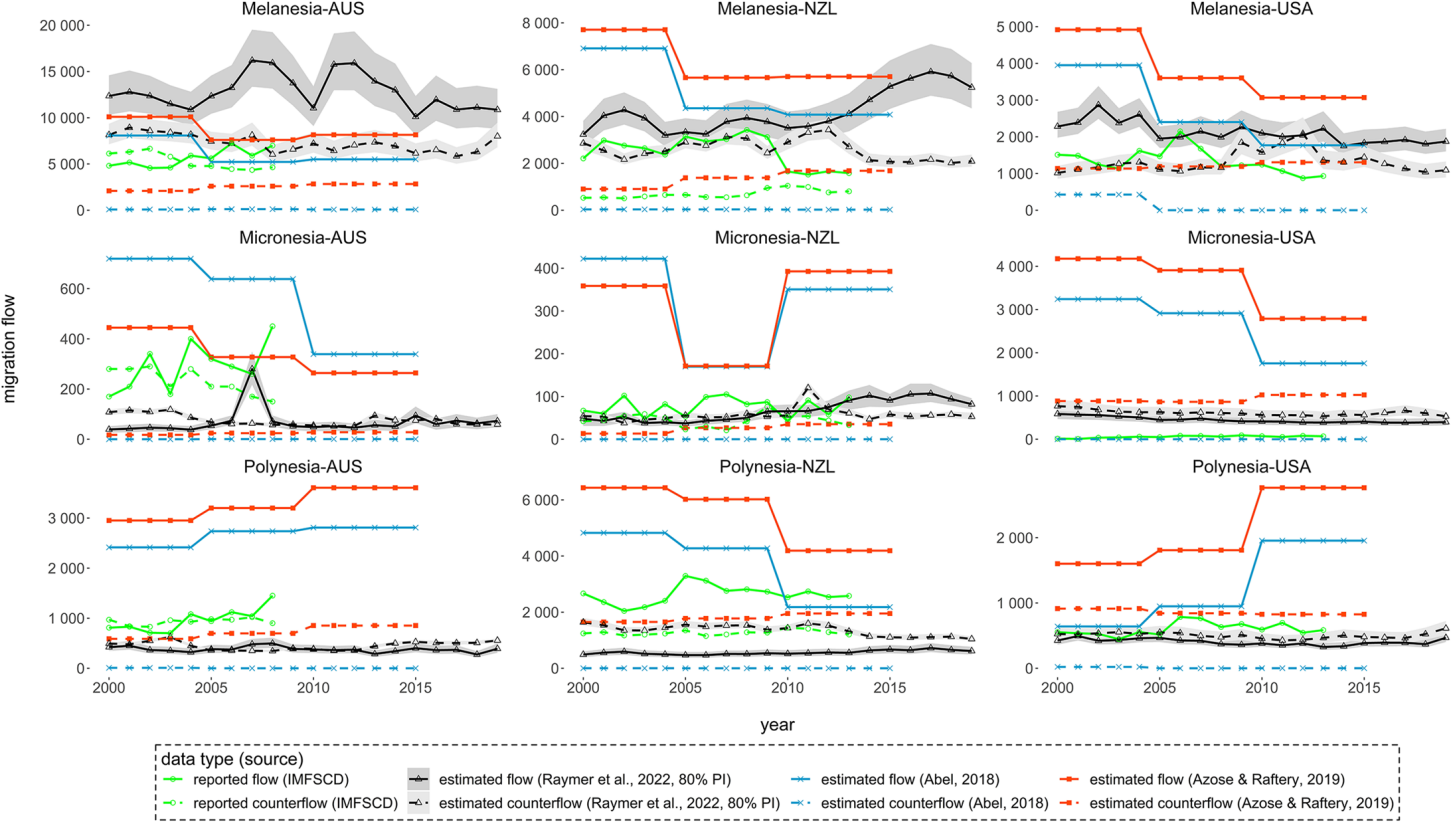
Reported and estimated migration flows between subregions in the Pacific and Australia (AUS), New Zealand (NZL) and the United States (USA): 2000–2019

Key migration corridors are examined in Fig. 2, highlighting the migration exchanges between three Pacific Islands subregions and three main receiving countries with historical colonial ties. The comparison between reported IMFSCD numbers and model-based estimates allows users to assess the performance of the three models against migration flow statistics collected by the major receiving countries. However, note that each country applies different definitions to produce statistics on migration (United Nations, 2015b).

In general, the reported levels of migration fall between the three sets of estimates. The exception is flows from Melanesia to New Zealand, where all three sets of estimates are higher than the reported numbers: 27–213% higher in Abel (2018), 65–275% higher in Azose and Raftery (2019), and 6–160% higher in Raymer et al. (2022). The facilitated access of migration to New Zealand for a number of Polynesian countries with colonial ties (Lee, 2009; Ware, 2005) is well represented in the IMFSCD data and model-based estimates in Fig. 2. Estimated flows from Pacific Island subregions to the United States are substantially higher than the reported statistics, albeit almost all countries in Micronesia have special access of migration to the United States (Lee, 2009; Ware, 2005). However, note that the measurement of immigrants in the United States’ data is considerably stricter than the United Nations’ recommended measure of migration being a change in the country of usual residence.

**Fig. 3:**
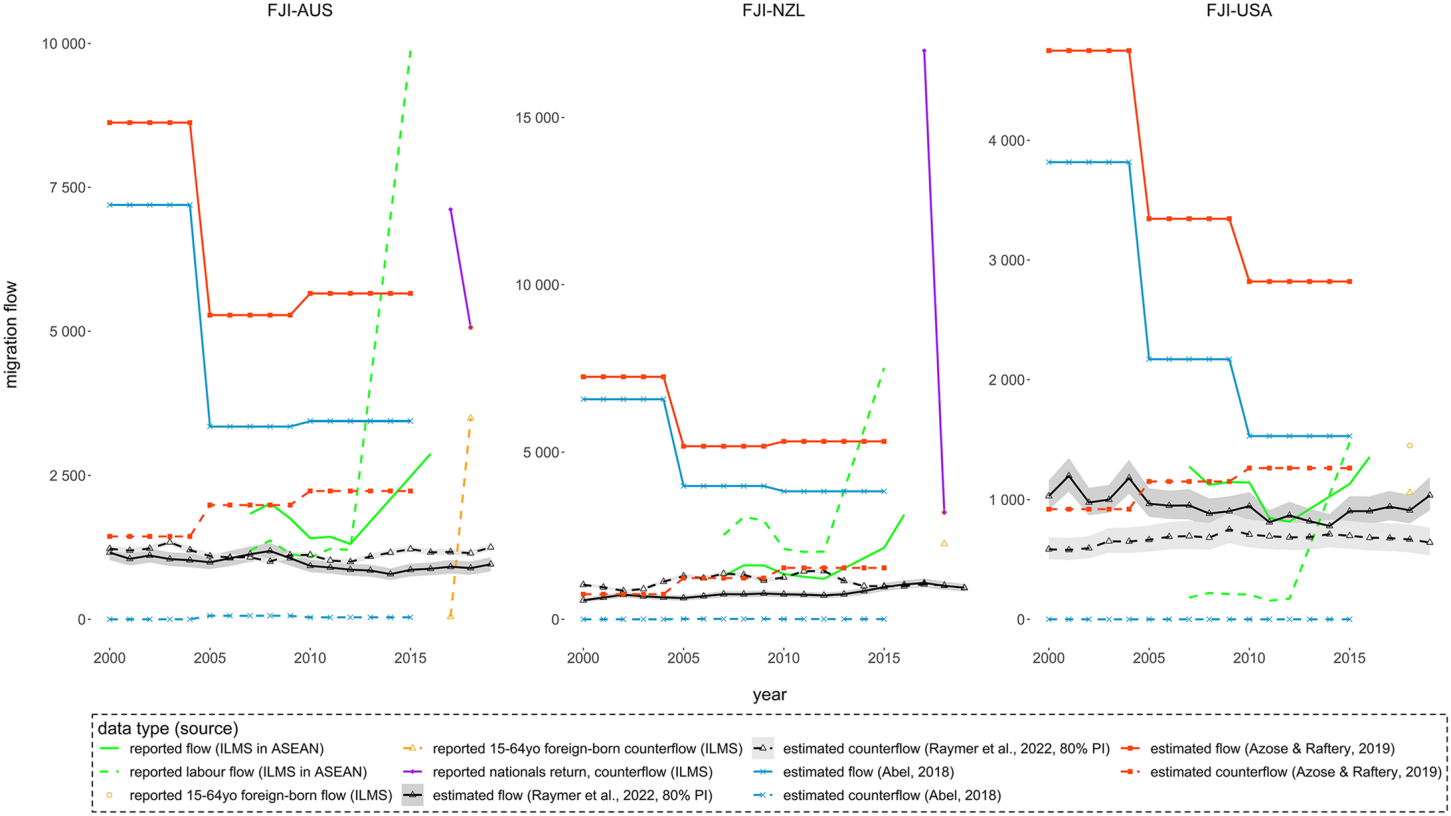
Reported and estimated migration flows between Fiji (FJI) and Australia (AUS), New Zealand (NZL) and the United States (USA): 2000–2019

**Fig. 4:**
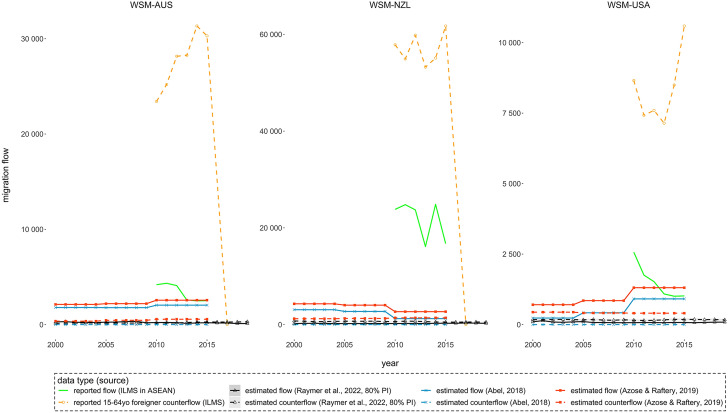
Reported and estimated migration flows between Samoa (WSM) and Australia (AUS), New Zealand (NZL) and the United States (USA): 2000–2019

Reported migration flow statistics for Fiji are plotted in Fig. 3 along with estimated flows and counterflows between Fiji and Australia, New Zealand and the United States. In Fig. 4, the reported and estimated flows and counterflows are presented for Samoa.

For Fiji, there are five types of reported flows available between 2000 and 2019 in ILMS databases. Reported emigration flows from Fiji to the three destination countries (solid green lines) fall within the range of the three model-based estimates and line up better with Raymer et al. (2022) before 2015. The post-2015 jumps in the reported statistics are attributed to that the data sources changed from the administrative records of citizens emigrating and citizens migrating for contractual employment in 2007–2012 to official estimates in 2015–2016 (ILO, 2015). Sourced from the same datasets, reported labour emigration flows from Fiji (dash green lines) were higher than reported emigration flows in some years, further problematizing the definitions of international migrants adopted by Fiji Bureau of Statistics. The large numbers of nationals returning from Australia and New Zealand reported by Fiji (solid purple lines) reflect the high circulation of migration activities. This is captured by correlated flows and counterflows in Raymer et al. (2022) but not by the flow-from-stock estimates.

Statistics reported by Samoa are substantially higher than what the three models estimated. The reported statistics are problematic with unclear duration and residence measurements on recorded migration. The reported emigration flows from Samoa (solid green lines) come from administrative records of annual international migration statistics but with no specified definition of emigrants. The reported working-age foreigner immigration flows (dash orange lines) before 2015 came from administrative records of non-citizens inflows, with no specified duration of stay of immigrants. The immigration flows suddenly dropped in 2017 for Australian and New Zealand citizens, with data coming from a different source, i.e. the Ministry of Commerce, Industry and Labour’s Employment Office records (ILO, 2015).

Comparisons can also be made between model-based estimates and reported visa statistics from Australia and New Zealand. Before the COVID-19 pandemic, the two largest Recognized Seasonal Employer and Seasonal Worker Programme sending countries are Tonga and Vanuatu (Curtain & Howes, 2020). Samoa recorded around 2000 Recognized Seasonal Employer visa recipients in the 2018–2019 financial year, a level comparable to the model-based estimates in Fig. 4. Their duration of stay, however, does not necessarily qualify them as long-term international migrants who have changed the country of usual residence.

## Discussion

International migration from, to and among Pacific Island countries is important and their importance is expected to increase in the future considering the region’s population growth, existing migration networks and rising environmental pressures. Data on the levels of migration from and to Pacific Island countries are required to better support and protect international migrants. Researchers and policymakers need to understand how migration in the Pacific Island region is evolving not only through qualitative approaches (e.g., International Migration Institute & University of Waikato, 2013) but also using quantitative tools. With no high-quality migration statistics currently in place for Pacific Island countries, this research has discussed and presented the possibility of using model-based estimates to improve our understanding of the levels of migration in the Pacific Island region. In doing so, we described the patterns resulting from three sets of model-based estimates (Abel, 2018; Azose & Raftery, 2019; Raymer et al., 2022).

Improved data collection and infrastructure are needed in the Pacific Island region. In the absence of reported data, estimates of Pacific Island migration flows may be used to form and test expectations regarding migration patterns. In this research brief, we demonstrated the advantages of using model-based estimates in comparing relative levels of migration flows across countries. While the estimated patterns are likely to contain error, they do provide a starting point for discussion and enquiry. Furthermore, as they are derived from models, enquiries of the estimates could be used to make further improvements to the methodology in the future. Without data or estimates, we have no means of enquiry and can only speculate how migration is occurring in the region.

Estimates may also provide the basis for devising migration regulations and providing support and protection to migrants. By having a detailed account of migration flows over time, we can understand more clearly how migrant groups are integrating and evolving across Pacific Island societies. For example, some migrant groups may exhibit particular demographic patterns or be geographically isolated. Having such evidence is useful for designing social policies directed at migrants and for conveying information to origin communities. Finally, estimates can be used to highlight where efforts should be placed to gather migration data and can provide a means for assessing data that are collected.

## Appendix

Table A1. Pacific Island countries by subregions

**Table:**

Subregion	Country code	Country name
Melanesia	FJI	Fiji
	NCL	New Caledonia
	PNG	Papua New Guinea
	SLB	Solomon Islands
	VUT	Vanuatu
Micronesia	FSM	The Federated States of Micronesia
	GUM	Guam
	KIR	Kiribati
	MHL	Marshall Islands
	MNP	Northern Mariana Islands
	NRU	Nauru
	PLW	Palau
Polynesia	ASM	American Samoa
	COK	Cook Islands
	NIU	Niue
	PYF	French Polynesia
	TKL	Tokelau
	TON	Tonga
	TUV	Tuvalu
	WLF	Wallis and Futuna Islands
	WSM	Samoa

## Footnotes

1 Refer to the Appendix for the list of Pacific Island countries by subregion.

2 For the discussion on difference between migration flow statistics and migrant population (stock) data, see “Migration Stocks and Flows” in Bartram et al. (2014)’s book “Key Concepts in Migration”.

3 Abel (2018) also used other versions of United Nations migrant stock data and World Bank data as inputs for alternative model estimates. What presented in this research brief are his estimates based on United Nations (2015c) version migrant stock data.
